# Central posterior capsule pigmentation in a patient with pigment dispersion and previous ocular trauma: A case report

**DOI:** 10.4103/0301-4738.64135

**Published:** 2010

**Authors:** Hani S Al-Mezaine

**Affiliations:** Department of Ophthalmology, College of Medicine, King Saud University, Riyadh, Saudi Arabia

**Keywords:** Pigment dispersion syndrome, pigmentation, posterior capsule, trauma

## Abstract

We report a 55-year-old man with unusually dense, unilateral central posterior capsule pigmentation associated with the characteristic clinical features of pigment dispersion syndrome, including a Krukenberg's spindle and dense trabecular pigmentation in both eyes. A history of an old blunt ocular trauma probably caused separation of the anterior hyaloid from the back of the lens, thereby creating an avenue by which pigment could reach the potential space of Berger's from the posterior chamber.

Pigment dispersion syndrome (PDS) is a well-known clinical entity usually discovered during routine examination of an otherwise healthy adult. This clinical condition, which is typically seen in younger, myopic males, is characterized by the liberation and deposition of melanin pigment granules on the corneal endothelium (Krukenberg's spindle) and the trabecular meshwork. It is also associated with radial defects of the iris pigment epithelium, which are visible by retroillumination during biomicroscopy. Pigment liberation is thought to occur secondary to the mechanical rubbing of the anterior lens zonules by the posterior iris surface.[1]

Blunt ocular trauma results in anteroposterior compression of the globe with simultaneous expansion in the equatorial plane. The impact of trauma is primarily absorbed by the lens-iris diaphragm and the vitreous base. Several changes have been described in the crystalline lens following blunt trauma, including contusion cataract, lens subluxation, and Vossius' ring.[2] In addition, the anterior hyaloid, which normally adheres firmly to the posterior lens capsule by the hyaloideocapsular ligament of Weiger, can detach following blunt trauma.[3] This is a case report of an unusual pattern of unilateral dense vitreo-lenticular pigment accumulation in an eye with history of blunt trauma in a patient with bilateral pigment dispersion.

## Case Report

**Figure 1:**
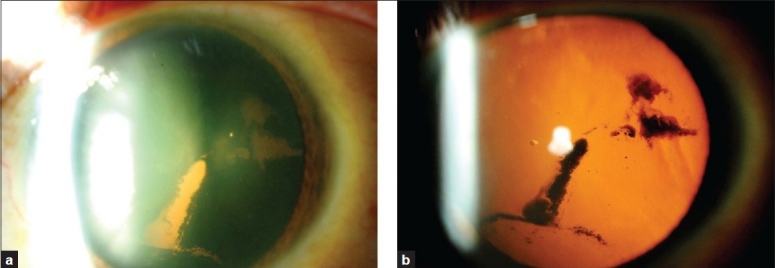
(a) a dense central patchy pigmentation of the posterior lens capsule of the left eye. (b) retroillumination of the same eye

**Figure 2:**
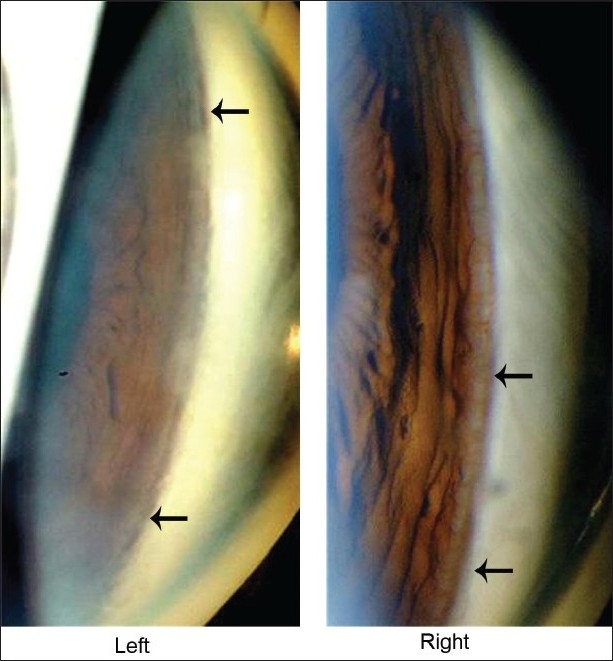
Gonioscopy showed trabecular meshwork hyperpigmentation (arrows) of both eyes

A 55-year-old man presented with complaint of floaters for one week. He gave a history of an old blunt trauma to the left eye by a football 15 years ago. The best corrected visual acuity was 20/20 in the right eye and 20/30 in the left eye. The subjective refraction was ‒5.50 diopter sphere (Dsph)‒0.75 D cylinder (cyl)×105 degrees in the right eye and ‒3.25 Dsph‒0.50 Dcyl×45 degrees in the left eye. The intraocular pressure was 14 mm Hg in both eyes. On slit-lamp examination, a pigment deposition on the endothelial side of the cornea (Krukenberg's spindle) was detected in both eyes; however, the iris examination was unremarkable. After pupillary dilatation, right eye examination revealed posterior vitreous detachment; however, lens, retina, and optic disc examinations were unremarkable. An examination of the left eye revealed a clear lens with a dense central patchy pigmentation of the posterior lens capsule [Fig. 1]. No other signs of blunt trauma were detected on detailed examination of the anterior and posterior segments of the left eye. Gonioscopy, performed prior to pupillary dilatation, showed open angles with hyperpigmentation of the trabecular meshwork of both eyes [Fig. 2].

## Discussion

In the present case, pigment dusting of the anterior iris surface was not observed because of the normal heavy pigmentation of the brown irides. In addition, iris transillumination defects were not present, which is typical for black patients with PDS.[4] However, the typical Krukenberg's spindle and the gonioscopic findings of homogenously heavy pigmentation of the trabecular meshwork were evident in this case. Interestingly, dense central posterior capsule pigmentation was also present in the left eye of the patient with PDS.

The hyaloideocapsular ligament of Weiger is an annular attachment of the anterior cortical vitreous to the posterior lens capsule. It is strongest in the midperipheral region of the lens. Although Weiger's ligament is normally invisible in the living eye, a prominent circular retrolental line corresponding to the Weiger's ligament has been observed biomicroscopically in the living eye in rare cases.[5] Just anterior and at the center of this ligament, two potential spaces are formed–sinus hyaloideocapsular and Berger's spaces, respectively. A posttraumatic peripheral annular pigment band on the posterior capsule (believed to be caused by melanin pigment scattering and deposition in the sinus hyaloideocapsular) has been reported.[2] Pigmentation of the peripheral lens capsule, classically in the shape of a line (Scheie's line[6] or Zentmayer's line[7]), has also been associated with PDS.

In this report, another type of posterior capsule pigmentation associated with PDS is described. It is characterized by dense pigmentation nearer to the posterior polar region of the lens, central to the position of Weiger's ligament (a potential space of Berger's) in a patient with a history of old blunt ocular trauma. To the best of my knowledge, the aqueous typically does not flow into a potential space of Berger's unless there is a probable defect on Weiger's ligament or anterior hyaloid detachment. Thus, central retrolental pigmentation could not be explained without a defect in these structures. This potential space may represent a dead space for pigment deposition, and is likely protected from normal aqueous currents and phagocytic cells. In the present case, history of old blunt trauma of the left eye probably caused this defect, thereby providing an avenue by which pigment could reach a potential space of Berger's from the posterior chamber. A brief report in the optometric literature described a minimal central retrolental pigmentation in two patients with PDS, with one patient having a history of mild ocular trauma.[6] Lin *et al*.[8] presented a case of dense pigmentation of the posterior lens capsule associated with PDS. However, in their patient, history of trauma was denied. In addition, Turgut *et al*.[9] described a case of non-traumatic annular and central heavy pigment deposition on the posterior lens capsule in PDS, and the central capsule pigmentation was attributed to a spontaneous detachment of the anterior hyaloid membrane.

In conclusion, this case showed that central posterior capsule pigmentation can be a presenting feature of PDS. However, an inquiry about the history of ocular trauma might explain the portal access whereby pigments reached the dead, inaccessible retrolental space.
